# (*E*)-*N*′-(3,5-Dibromo-2-hydroxy­benzyl­idene)nicotinohydrazide

**DOI:** 10.1107/S1600536810006276

**Published:** 2010-02-20

**Authors:** Yong-Qing Su, Cong Li, Ping Wang

**Affiliations:** a Faculty of Chemistry and Chemical Engineering, Yunnan Normal University, Kunming 650092, People’s Republic of China

## Abstract

In the title Schiff base compound, C_13_H_9_Br_2_N_3_O_2_, there is an intra­molecular O-H⋯N hydrogen bond involving the hydroxyl substituent and the adjacent hydrazine N atom. The mol­ecule is almost planar, the dihedral angle between the benzene ring and the pyridine ring being 5.7 (2)°. In the crystal structure, symmetry-related mol­ecules are linked *via* N—H⋯O hydrogen bonds, forming chains propagating in [001].

## Related literature

For related literature on Schiff bases, see: Archibald *et al.* (1994 ▶); Harada *et al.* (1999 ▶); Ogawa *et al.* (1998 ▶). For similar structures, see: Mohd Lair *et al.* (2009 ▶); Li *et al.* (2010 ▶); Sun *et al.* (2009 ▶); Wang *et al.* (2010 ▶); Wen *et al.* (2009 ▶).
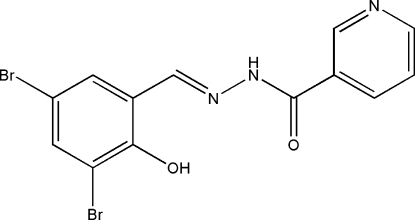


## Experimental

### 

#### Crystal data

C_13_H_9_Br_2_N_3_O_2_
*M* *_r_* = 399.05
Monoclinic, 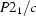

*a* = 17.013 (4) Å
*b* = 8.091 (2) Å
*c* = 10.153 (3) Å
β = 92.194 (13)°
*V* = 1396.6 (6) Å^3^
*Z* = 4
Mo *K*α radiation
μ = 5.81 mm^−1^
*T* = 298 K
0.23 × 0.21 × 0.20 mm

#### Data collection

Bruker APEXII CCD area-detector diffractometer
Absorption correction: multi-scan (*SADABS*; Sheldrick, 2004 ▶) *T*_min_ = 0.349, *T*_max_ = 0.390
8024 measured reflections
3023 independent reflections
1933 reflections with *I* > 2σ(*I*)
*R*_int_ = 0.036

#### Refinement

*R*[*F* ^2^ > 2σ(*F* ^2^)] = 0.038
*wR*(*F* ^2^) = 0.111
*S* = 1.00
3023 reflections
185 parameters
1 restraint
H atoms treated by a mixture of independent and constrained refinement
Δρ_max_ = 0.64 e Å^−3^
Δρ_min_ = −0.69 e Å^−3^

### 

Data collection: *APEX2* (Bruker, 2004 ▶); cell refinement: *SAINT* (Bruker, 2004 ▶); data reduction: *SAINT*; program(s) used to solve structure: *SHELXS97* (Sheldrick, 2008 ▶); program(s) used to refine structure: *SHELXL97* (Sheldrick, 2008 ▶); molecular graphics: *SHELXTL* (Sheldrick, 2008 ▶); software used to prepare material for publication: *SHELXTL*.

## Supplementary Material

Crystal structure: contains datablocks global, I. DOI: 10.1107/S1600536810006276/su2163sup1.cif

Structure factors: contains datablocks I. DOI: 10.1107/S1600536810006276/su2163Isup2.hkl

Additional supplementary materials:  crystallographic information; 3D view; checkCIF report

## Figures and Tables

**Table 1:** Hydrogen-bond geometry (Å, °)

*D*—H⋯*A*	*D*—H	H⋯*A*	*D*⋯*A*	*D*—H⋯*A*
O1—H1⋯N1	0.82	1.89	2.609 (4)	146
N2—H2⋯O2^i^	0.91 (2)	2.14 (2)	3.017 (4)	162 (3)

Symmetry code: (i) 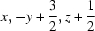.

**Fig. 1.**
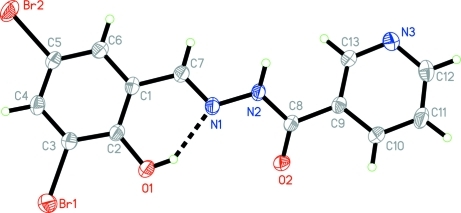
The molecular structure of the title compound, showing the atom-numbering scheme. Displacement ellipsoids are drawn at the 30% probability level. The intramolecular O-H···N hydrogen bond is shown as a dashed line.

**Fig. 2.**
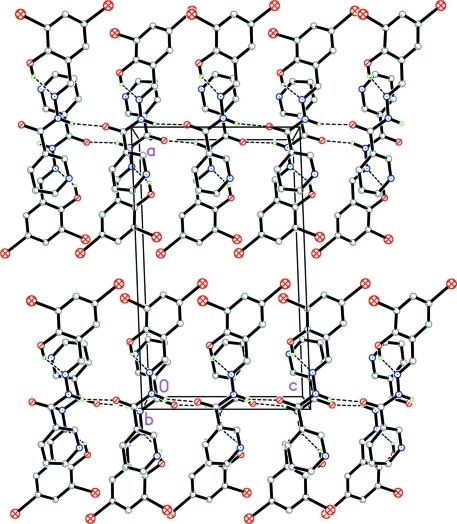
The crystal packing of the title compound viewed along the b-axis. The intra- and intermolecular hydrogen bonds are shown as dashed lines (see Table 1 for details).

## supplementary crystallographic information

### Comment

Schiff bases have received much attention in recent years (Ogawa *et al.*, 1998; Archibald *et al.*, 1994; Harada *et al.*, 1999). Recently, we reported on the crystal structures of two new Schiff bases (Li *et al.*, 2010; Wang *et al.*, 2010). As a further investigation of the structures of Schiff base compounds the title compound was prepared by the reaction of 3,5-dibromo-2-hydroxybenzaldehyde with nicotinic acid hydrazide in methanol; its crystal structure is reported on here.

In the title compound (Fig. 1), there is an intramolecular O-H···N hydrogen bond involving the hydroxyl substituent and the adjacent hydrazine N-atom, N2 (Table 1). The benzene ring is inclined to the pyridine ring by 5.7 (2)°. All the bond lengths are comparable with those observed in similar Schiff bases reported on previously (Wen *et al.*, 2009; Mohd Lair *et al.*, 2009; Sun *et al.*, 2009).

In the crystal structure, symmetry related molecules are linked via intermolecular N—H···O hydrogen bonds so forming chains running along the *c* axis (Table 1, Fig. 2).

### Experimental

3,5-Dibromo-2-hydroxybenzaldehyde (1.0 mmol, 280 mg) and nicotinic acid hydrazide (1.0 mmol, 137 mg) were dissolved in methanol (30 mL). The mixture was stirred at room temperature for about 1 h to give a colorless solution. After allowing the solution to evaporate slowly in air for 8 days, colorless block-like crystals, suitable for X-ray analysis, were formed.

### Refinement

Atom H2 was located in a difference Fourier map and refined with a N—H distance restraint of 0.90 (1) Å and U_iso_(H) = 0.08. The other H atoms were placed in idealized positions and constrained to ride on their parent atoms: O—H = 0.82 Å, C—H = 0.93 Å, with U_iso_(H) = k × U_eq_(parent atom), where k = 1.2 for C-bound H-atoms and = 1.5 for the hydroxyl H-atom.

### Crystal data

**Table:**

C_13_H_9_Br_2_N_3_O_2_	*F*(000) = 776
*M**_r_* = 399.05	*D*_x_ = 1.898 Mg m^−^^3^
Monoclinic, *P*2_1_/*c*	Mo *K*α radiation, λ = 0.71073 Å
Hall symbol: -P 2ybc	Cell parameters from 2084 reflections
*a* = 17.013 (4) Å	θ = 2.4–25.2°
*b* = 8.091 (2) Å	µ = 5.81 mm^−^^1^
*c* = 10.153 (3) Å	*T* = 298 K
β = 92.194 (13)°	Block, colourless
*V* = 1396.6 (6) Å^3^	0.23 × 0.21 × 0.20 mm
*Z* = 4	

### Data collection

**Table:**

Bruker APEXII CCD area-detector diffractometer	3023 independent reflections
Radiation source: fine-focus sealed tube	1933 reflections with *I* > 2σ(*I*)
graphite	*R*_int_ = 0.036
ω scans	θ_max_ = 27.0°, θ_min_ = 1.2°
Absorption correction: multi-scan (*SADABS*; Sheldrick, 2004)	*h* = −19→21
*T*_min_ = 0.349, *T*_max_ = 0.390	*k* = −10→10
8024 measured reflections	*l* = −12→8

### Refinement

**Table:**

Refinement on *F*^2^	Primary atom site location: structure-invariant direct methods
Least-squares matrix: full	Secondary atom site location: difference Fourier map
*R*[*F*^2^ > 2σ(*F*^2^)] = 0.038	Hydrogen site location: inferred from neighbouring sites
*wR*(*F*^2^) = 0.111	H atoms treated by a mixture of independent and constrained refinement
*S* = 1.00	*w* = 1/[σ^2^(*F*_o_^2^) + (0.0589*P*)^2^] where *P* = (*F*_o_^2^ + 2*F*_c_^2^)/3
3023 reflections	(Δ/σ)_max_ = 0.001
185 parameters	Δρ_max_ = 0.64 e Å^−^^3^
1 restraint	Δρ_min_ = −0.69 e Å^−^^3^

### Special details

**Table:**

Geometry. All esds (except the esd in the dihedral angle between two l.s. planes) are estimated using the full covariance matrix. The cell esds are taken into account individually in the estimation of esds in distances, angles and torsion angles; correlations between esds in cell parameters are only used when they are defined by crystal symmetry. An approximate (isotropic) treatment of cell esds is used for estimating esds involving l.s. planes.
Refinement. Refinement of *F*^2^ against ALL reflections. The weighted *R*-factor wR and goodness of fit *S* are based on *F*^2^, conventional *R*-factors *R* are based on *F*, with *F* set to zero for negative *F*^2^. The threshold expression of *F*^2^ > σ(*F*^2^) is used only for calculating *R*-factors(gt) etc. and is not relevant to the choice of reflections for refinement. *R*-factors based on *F*^2^ are statistically about twice as large as those based on *F*, and *R*- factors based on ALL data will be even larger.

### Fractional atomic coordinates and isotropic or equivalent isotropic displacement parameters (Å^2^)

**Table:**

	*x*	*y*	*z*	*U*_iso_*/*U*_eq_	
Br1	0.38611 (2)	1.15620 (7)	0.38681 (5)	0.0731 (2)	
Br2	0.44353 (2)	0.85756 (6)	0.88313 (4)	0.06341 (18)	
N1	0.11733 (17)	0.8462 (3)	0.5380 (3)	0.0393 (7)	
N2	0.04257 (16)	0.7852 (4)	0.5483 (3)	0.0402 (7)	
N3	−0.16645 (18)	0.5160 (4)	0.5780 (3)	0.0501 (8)	
O1	0.22622 (14)	1.0061 (3)	0.4163 (2)	0.0514 (7)	
H1	0.1820	0.9696	0.4270	0.077*	
O2	0.01730 (14)	0.8169 (3)	0.3293 (2)	0.0508 (7)	
C1	0.2459 (2)	0.8814 (4)	0.6310 (3)	0.0383 (8)	
C2	0.27257 (19)	0.9725 (4)	0.5231 (3)	0.0377 (8)	
C3	0.3494 (2)	1.0292 (4)	0.5283 (3)	0.0437 (9)	
C4	0.4001 (2)	0.9964 (4)	0.6347 (3)	0.0455 (9)	
H4	0.4512	1.0374	0.6373	0.055*	
C5	0.3737 (2)	0.9026 (4)	0.7363 (3)	0.0403 (8)	
C6	0.2980 (2)	0.8450 (4)	0.7371 (3)	0.0430 (9)	
H6	0.2813	0.7823	0.8076	0.052*	
C7	0.1655 (2)	0.8224 (4)	0.6360 (3)	0.0396 (8)	
H7	0.1490	0.7673	0.7104	0.048*	
C8	−0.00388 (19)	0.7712 (4)	0.4372 (3)	0.0366 (8)	
C9	−0.08166 (19)	0.6936 (4)	0.4557 (3)	0.0358 (8)	
C10	−0.1424 (2)	0.7215 (5)	0.3642 (4)	0.0492 (9)	
H10	−0.1347	0.7889	0.2917	0.059*	
C11	−0.2145 (2)	0.6488 (5)	0.3811 (4)	0.0541 (11)	
H11	−0.2561	0.6666	0.3207	0.065*	
C12	−0.2236 (2)	0.5493 (5)	0.4894 (4)	0.0510 (10)	
H12	−0.2727	0.5025	0.5011	0.061*	
C13	−0.0972 (2)	0.5869 (4)	0.5595 (3)	0.0417 (9)	
H13	−0.0563	0.5637	0.6200	0.050*	
H2	0.024 (2)	0.764 (6)	0.629 (2)	0.080*	

### Atomic displacement parameters (Å^2^)

**Table:**

	*U*^11^	*U*^22^	*U*^33^	*U*^12^	*U*^13^	*U*^23^
Br1	0.0414 (3)	0.1045 (4)	0.0730 (3)	−0.0128 (2)	−0.0014 (2)	0.0354 (3)
Br2	0.0494 (3)	0.0779 (3)	0.0609 (3)	0.0041 (2)	−0.0244 (2)	0.0057 (2)
N1	0.0304 (16)	0.0528 (18)	0.0347 (16)	−0.0058 (13)	0.0011 (12)	−0.0056 (13)
N2	0.0285 (16)	0.0596 (19)	0.0324 (16)	−0.0048 (14)	−0.0016 (12)	−0.0034 (14)
N3	0.0425 (19)	0.057 (2)	0.0510 (19)	−0.0091 (15)	0.0009 (15)	0.0027 (15)
O1	0.0355 (15)	0.079 (2)	0.0393 (14)	−0.0054 (13)	−0.0059 (11)	0.0100 (13)
O2	0.0404 (15)	0.082 (2)	0.0301 (14)	−0.0050 (13)	−0.0007 (11)	0.0079 (13)
C1	0.0308 (19)	0.044 (2)	0.040 (2)	−0.0013 (15)	−0.0025 (15)	−0.0067 (16)
C2	0.0319 (19)	0.047 (2)	0.0344 (18)	0.0030 (15)	−0.0022 (14)	−0.0012 (16)
C3	0.035 (2)	0.051 (2)	0.045 (2)	−0.0012 (16)	−0.0013 (16)	0.0044 (17)
C4	0.030 (2)	0.051 (2)	0.055 (2)	−0.0002 (17)	−0.0051 (17)	−0.0015 (19)
C5	0.036 (2)	0.044 (2)	0.041 (2)	0.0035 (16)	−0.0078 (15)	−0.0012 (17)
C6	0.043 (2)	0.047 (2)	0.039 (2)	0.0002 (17)	−0.0033 (16)	−0.0008 (16)
C7	0.037 (2)	0.044 (2)	0.037 (2)	−0.0012 (15)	−0.0033 (16)	−0.0010 (15)
C8	0.0315 (19)	0.043 (2)	0.0347 (19)	0.0033 (15)	−0.0029 (15)	−0.0034 (16)
C9	0.0322 (19)	0.043 (2)	0.0318 (18)	0.0028 (15)	−0.0015 (14)	−0.0087 (15)
C10	0.042 (2)	0.066 (3)	0.038 (2)	−0.0023 (19)	−0.0067 (17)	0.0071 (19)
C11	0.034 (2)	0.077 (3)	0.050 (2)	0.0003 (19)	−0.0100 (17)	−0.011 (2)
C12	0.037 (2)	0.056 (2)	0.061 (3)	−0.0079 (18)	0.0017 (18)	−0.012 (2)
C13	0.038 (2)	0.047 (2)	0.039 (2)	−0.0013 (17)	−0.0042 (16)	0.0019 (16)

### Geometric parameters (Å, °)

**Table:**

Br1—C3	1.892 (4)	C3—C4	1.382 (4)
Br2—C5	1.905 (3)	C4—C5	1.370 (5)
N1—C7	1.278 (4)	C4—H4	0.9300
N1—N2	1.372 (4)	C5—C6	1.371 (5)
N2—C8	1.357 (4)	C6—H6	0.9300
N2—H2	0.901 (10)	C7—H7	0.9300
N3—C12	1.326 (4)	C8—C9	1.483 (5)
N3—C13	1.330 (4)	C9—C10	1.381 (5)
O1—C2	1.344 (4)	C9—C13	1.396 (5)
O1—H1	0.8200	C10—C11	1.377 (5)
O2—C8	1.223 (4)	C10—H10	0.9300
C1—C6	1.401 (5)	C11—C12	1.376 (5)
C1—C2	1.409 (5)	C11—H11	0.9300
C1—C7	1.452 (5)	C12—H12	0.9300
C2—C3	1.384 (5)	C13—H13	0.9300
			
C7—N1—N2	117.1 (3)	C1—C6—H6	120.3
C8—N2—N1	118.6 (3)	N1—C7—C1	120.0 (3)
C8—N2—H2	122 (3)	N1—C7—H7	120.0
N1—N2—H2	119 (3)	C1—C7—H7	120.0
C12—N3—C13	116.5 (3)	O2—C8—N2	122.5 (3)
C2—O1—H1	109.5	O2—C8—C9	122.4 (3)
C6—C1—C2	119.6 (3)	N2—C8—C9	115.1 (3)
C6—C1—C7	118.3 (3)	C10—C9—C13	116.7 (3)
C2—C1—C7	122.1 (3)	C10—C9—C8	119.6 (3)
O1—C2—C3	119.2 (3)	C13—C9—C8	123.6 (3)
O1—C2—C1	122.4 (3)	C11—C10—C9	119.6 (4)
C3—C2—C1	118.4 (3)	C11—C10—H10	120.2
C4—C3—C2	121.7 (3)	C9—C10—H10	120.2
C4—C3—Br1	118.9 (3)	C12—C11—C10	118.5 (4)
C2—C3—Br1	119.4 (3)	C12—C11—H11	120.8
C5—C4—C3	118.9 (3)	C10—C11—H11	120.8
C5—C4—H4	120.6	N3—C12—C11	124.0 (4)
C3—C4—H4	120.6	N3—C12—H12	118.0
C4—C5—C6	121.9 (3)	C11—C12—H12	118.0
C4—C5—Br2	118.9 (3)	N3—C13—C9	124.6 (3)
C6—C5—Br2	119.2 (3)	N3—C13—H13	117.7
C5—C6—C1	119.4 (3)	C9—C13—H13	117.7
C5—C6—H6	120.3		

### Hydrogen-bond geometry (Å, °)

**Table:**

*D*—H···*A*	*D*—H	H···*A*	*D*···*A*	*D*—H···*A*
O1—H1···N1	0.82	1.89	2.609 (4)	146
N2—H2···O2^i^	0.91 (2)	2.14 (2)	3.017 (4)	162 (3)

Symmetry codes: (i) *x*, −*y*+3/2, *z*+1/2.
